# What Are the Molecular Mechanisms by Which Functional Bacterial Amyloids Influence Amyloid Beta Deposition and Neuroinflammation in Neurodegenerative Disorders?

**DOI:** 10.3390/ijms21051652

**Published:** 2020-02-28

**Authors:** Robert P. Friedland, Joseph D. McMillan, Zimple Kurlawala

**Affiliations:** 1 Department of Neurology, University of Louisville School of Medicine, Louisville, KY 40202, USA; 2 Department, University of South Florida, Tampa, FL 33620, USA; jmcmillan3@health.usf.edu; 3 National Institute on Aging, Bethesda, MD 33620, USA; zimple.kurlawala@louisville.edu

**Keywords:** Alzheimer’s disease, microbiota, bacterial amyloid, FUBA, curli, CsgA, amyloid beta, neuroinflammation

## Abstract

Despite the enormous literature documenting the importance of amyloid beta (Aβ) protein in Alzheimer’s disease, we do not know how Aβ aggregation is initiated and why it has its unique distribution in the brain. In vivo and in vitro evidence has been developed to suggest that functional microbial amyloid proteins produced in the gut may cross-seed Aβ aggregation and prime the innate immune system to have an enhanced and pathogenic response to neuronal amyloids. In this commentary, we summarize the molecular mechanisms by which the microbiota may initiate and sustain the pathogenic processes of neurodegeneration in aging.

## 1. Introduction

The original descriptions of cortical plaques in subjects with dementia was provided by several scientists, including Emil Redlich (1898), Koichi Miyake (1906), Alois Alzheimer (1906), Oskar Fischer (1907), and Soloman Carter Fuller (1907) [1]. However, the molecular analysis of the material in the plaque was not clarified until Glenner and Wong (1984) described the amino acid sequence of the amyloid which they found to have no homology to any known protein. Subsequently, it has been determined that the protein, now called amyloid Beta (Aβ), is a breakdown product of a larger molecule, the amyloid precursor protein (APP), coded by a gene on chromosome 21. Mutations in APP have been found to be a genetic cause of early-onset Alzheimer’s disease (AD). This led to the “amyloid hypothesis”, which posited that accumulation of the Aβ protein in the brain is responsible for the pathogenesis of AD [2,3].

There is now overwhelming evidence that Aβ buildup in the brain is certainly an important part of the AD pathogenic process. But it remains to be determined if it is the critical factor responsible. Furthermore, the amyloid hypothesis does not explain how the process starts. That is, what is the initiating factor responsible for Aβ accumulation, and why do some people get the disease and others remain unaffected? This is a critical question in the ~99% of cases of AD that are sporadic, and do not have a causative gene.

Prusiner has suggested that the key mechanism in Alzheimer’s etiology is stochastic—an unfortunate misfolding of the Aβ protein causing a non-catalytic, prion-like self-replicating pathogenic process [4]. We find the stochastic explanation unsatisfactory and prefer to look for the opportunity for environmental factors to be involved. Our largest environmental exposure is to the myriad organisms (bacteria, viruses, archaea, yeasts, parasites, fungi) which reside on our body surfaces and in our body cavities. It has now been extensively documented that more than half of a person’s cells are of microbial origin, and there are 100 times more nucleotide sequences in our bodies coding for bacterial, rather than human proteins [5]. In this commentary, we will examine the potential molecular mechanisms by which the microbiota may influence cortical Aβ deposition. The mechanisms noted below may operate independently, or more likely, work in unison (i.e, neuroinflammation triggers Aβ aggregation [6]).

## 2. Functional Bacterial Amyloid Proteins (FUBA) May Cross-Seed the Aggregation of Aβ as Well as Other Neuronal Amyloids

Chapman and colleagues originally described amyloid proteins made by bacteria in 2002 [7]. It has now been demonstrated that many organisms present inside our bodies are capable of producing FUBAs, including Streptococcus, Staphylococcus, Salmonella, Mycobacteria, Klebsiella, Citrobacter, and Bacillus species [8]. These bacterial amyloids are adaptive, help the organisms stick together, and protect bacterial communities by resisting destruction by viruses and other agents. Bacterial amyloids have been linked to infections as well to autoimmunity [9]. In 2015, Friedland proposed that bacterial amyloid may cross-seed the aggregation of neuronal proteins such as Aβ, alpha synuclein (AS), tau and others to initiate a prion-like propagation [10]. This process may begin with enteroendocrine and M cells in the gut epithelium and be transmitted to the brain through the autonomic nervous system via a bidirectional pathway (the gut-brain axis, [11,12]). It was subsequently demonstrated by Chen et al. that feeding bacterial amyloid to aged rats accelerates AS deposition in the gut, as well as in the brain [13].

It has been observed that protein folding in an amyloid configuration is a highly conserved process throughout evolution [14]. Recently, a series of studies have shown that FUBAs may interact with neuronal proteins in the manner predicted. That is, a bacterial amyloid protein may influence the aggregation of neuronal proteins. Perov et al. discovered structural similarity between the fibers of the best studied bacterial amyloid protein curli, and neuronal amyloids [14]. They noted that the curli protein CsgA cross-seeds the fibrillation of Aβ, as was previously proposed [10]. Curli cross-seeding with Aβ was also observed to have a concentration dependent effect, suggesting that this cross-seeding may be of widespread impact [15]. It has also been shown that the functional amyloid protein FapC, produced by Pseudomonas, may affect fibrillation of AS [16]. It should be noted that these studies were done in vitro with nonbiological buffers, suggesting a need for in vivo studies using simple systems such as yeast or *C. elegans* to demonstrate this process.

## 3. FUBAs May Enhance Neuro-Inflammation through Molecular Mimicry

Gut bacteria have been shown to remarkably influence microglial function [17] and bacterial amyloid is recognized by the innate immune system as a pathogen associated molecular pattern (PAMP) involving toll-like receptor 2 (TLR2), cluster of differentiation 14 (CD14), nuclear factor kappa light chain enhancer of activated B cells (NFkB), and inducible nitric oxide synthase (iNOS) [10]. Remarkably, this pathway is also involved in the recognition of misfolded neuronal proteins such as Aβ and AS [18]. The presence of Aβ aggregates in the brain, often found with healthy aging, may be recognized by the innate immune system as a bacterial product, due to its structural similarity to bacterial proteins. Bacterial amyloid also causes activation of the NLRP3 inflammasome, causing downstream release of pro-inflammatory interleukin-1β in microglia, eventually leading to Aβ and tau aggregation [6,19,20].

## 4. Microbiota May Influence Inflammation in the Brain through Effects on Circulating Immune Cells

Over 70% of our lymphocytes reside in the gut—a sign of the enormous influence of the microbiota on the immune system. Bacteria produce short-chain fatty acids such as propionate, butyrate and acetate that epigenetically promote the function of anti-inflammatory regulatory lymphocytes (Tregs) [21]. Conversely, some groups of bacteria enhanced by a low fiber diet do not produce short chain fatty acids and upregulate the production of pro-inflammatory CD4+ Th17 cells [21]. As we have discussed above, there is an inflammatory component to all neurodegenerations.

## 5. Microbiota May Influence the Effects of Apolipoprotein E (Apo E) Genotype on Disease Risk

It was proposed in 2015 that the effect of Apo E on Alzheimer risk may be caused by the influence of Apo E genotype on the microbiota [10]. Recently, Tran et al. provided evidence that in humans and transgenic mice the microbial populations and metabolite panels are different between ApoE genotypes [22]. This relationship may provide a valuable clue to development of microbiota-based therapies. It is important to recall that susceptibility to Creutzfeldt Jakob disease and bovine spongiform encephalopathy is determined by the genotype at codon 129 of the prion protein gene (people who are heterozygous MV, or valine homozygotes VV are resistant).

## 6. Microbiota May Influence Neurodegeneration through Production of Vitamins and Other Nutrients

The microbiome produce important metabolic products that influence health and disease. It was recently demonstrated that gut bacteria are involved in amyotrophic lateral sclerosis (ALS). Transgenic ALS hSOD1 G93A mice were found to have bacterial dysbiosis and mice lacking gut bacteria had accelerated disease. Preliminary studies suggest that it may be due to a deficiency of nicotinamide (vitamin B3) [23]. Nicotinamide is a NAD+ precursor crucial for energy and redox metabolism [24].

## 7. Further Considerations

Current microbiota studies have focused largely on bacterial populations in the intestines involving studies of stool. The potential role of oral, nasal, laryngeal and pharyngeal organisms has not been extensively studied. This is especially important in neurodegeneration, as hyposmia is a feature of both AD and Parkinson’s disease (PD). Furthermore, the proximity of the brain to the nose (which has its own microbiota) demonstrates the opportunity for interactions between olfactory receptors, microbes and microbial metabolites [25,26]. The mouth is also a critical ecological niche for hundreds of different bacterial species in humans. Also, the common oral pathogen *Streptococcus mutans* may produce an amyloid product. Studies of stool do not assess the intestinal microbiota that reside in the mucus layer. In addition to the description of the populations present in the gut, it is imperative to consider metabolic products generated by the microbiota, which undoubtedly influence health and disease [27,28].

A central question which remains unanswered is: what FUBAs are present in the body, and how much? Current work has been devoted largely to bacteria while the role of viruses, fungi, parasites, and other agents have not been considered. It is estimated that there may be twice as many phagesin the gut as bacteria—their contribution to bacterial homeostasis is just beginning to be addressed. Furthermore, fungi can act as commensals in the gut and a few fungal amyloid proteins have been described [29]. Additional variables that need to be considered are sex-specific microbiota changes, as recently demonstrated in antibiotic treated transgenic mouse models of Aβ amyloidosis [30], and their potential effect of sex on FUBAs. These outstanding questions are addressed in the Table 1.

One potentially exciting aspect to microbiota studies is the relative ease with which microbial populations can be adjusted. This can be called “gene therapy in the kitchen”, because it is possible to change bacterial populations in the gut by changing diet in as little as two weeks [31]. The potential for therapeutic interventions based on FUBA is illustrated by a recent paper by Sampson et al. [32] that elegantly reports the role of the curli (CsgA) protein in producing neurodegeneration in alpha synuclein overexpressing transgenic PD model mice. Oral treatment of wild type *E. coli* producing the bacterial amyloid protein CsgA with epigallocatechin gallate (EGCG) reduced biofilm formation and inhibited the production of aggregated alpha synuclein. EGCG is a potent polyphenol found in plants, which impairs the production of bacterial CsgA. As EGCG is not well-absorbed from the gut to the bloodstream, it is likely that these influences are localized to the intestinal microbiota. This work supports the exciting opportunity to treat neurodegenerative diseases with agents that act primarily on the microbiota, and not directly on the brain. The potential for influencing pathogenic processes in AD through therapies targeting the gut shows great promise and is now receiving academic and commercial attention worldwide.

## Figures and Tables

**Table 1:** Outstanding questions concerning the role of functional bacterial amyloid proteins (FUBA) and neurodegeneration.

Which organisms are producing these FUBA?
Is there a basal *normal* level of FUBA in the gut?
Are there unique niches for the production of FUBA? (nose, mouth, pharynx, larynx, stomach, duodenum, Ilium, appendix, colon, skin, external ear, elsewhere)
Are there strains of FUBA which interact with specific neurodegenerative disease proteins involved in AD, PD, ALS, progressive supranuclear palsy, cortical basal degeneration, multiple system atrophy?
Can the production of FUBA be regulated by diet and drugs?
Do phages, fungi and archaea influence the production of FUBA?
What are the effects of FUBA on metabolic products of the microbiota?
How does sex influence FUBA and their effects?
Are functional amyloids produced by non-bacterial species like fungi involved in human diseases?
